# Estimating the prevalence of researcher misconduct: a study of UK academics within biological sciences

**DOI:** 10.7717/peerj.562

**Published:** 2014-09-09

**Authors:** David L. Roberts, Freya A.V. St. John

**Affiliations:** Durrell Institute of Conservation and Ecology, School of Anthropology and Conservation, University of Kent, Canterbury, Kent, UK

**Keywords:** Unmatched-count technique, Crosswise-model, Sensitive question, Ethics, List experiment

## Abstract

Misconduct in academic research is undoubtedly increasing, but studies estimating the prevalence of such behaviour suffer from biases inherent in researching sensitive topics. We compared the unmatched-count technique (UCT) and the crosswise-model (CM), two methods specifically designed to increase honest reporting to sensitive questions, with direct questioning (DQ) for five types of misconduct in the biological sciences. UCT performed better than CM and either outperformed or produced similar estimates to DQ depending on the question. Estimates of academic misconduct increased with decreasing seriousness of the behaviour, from *c*. 0% for data fabrication to >68% for inappropriate co-authorship. Results show that research into even minor issues of misconduct, is sensitive, suggesting that future studies should consider using specialised questioning techniques as they are more likely to yield accurate figures.

## Introduction

Misconduct by academics is reportedly increasing (Steen, Casadevall & Fang, 2013), and known cases represent only the “tip-of-the-iceberg” (Fanelli, 2009). However, not all categories of misconduct are equally reprehensible. A continuum exists from ideal research behaviour, through questionable practices, to the most serious categories: fabrication, falsification, and plagiarism (Steneck, 2006)—the focus of most research into misconduct. However, even minor infractions should not be taken lightly as they can lead to self-justification of misconduct (Casadevall & Fang, 2012). Deviant research behaviour is not only detrimental to the individual, but also to society, as fraudulent research can misdirect future research, funding and policy. It also disadvantages compliant academics and, when unchecked, can result in a perception that the easiest way to progress in academia is to cheat (Casadevall & Fang, 2012).

Research misconduct is socially unacceptable, therefore estimating its prevalence is challenging. To date, studies have taken two forms. The first form encompasses the analysis of types of fraud reported to offices for research integrity (e.g., Fang, Bennett & Casadevall, 2013) or of causes of retractions from academic journals (e.g., Fang, Steen & Casadevall, 2012; Steen, Casadevall & Fang, 2013); such studies do not provide an estimate of prevalence. The second form is through direct questions, asking respondents about their own involvement in such activities and/or to estimate the prevalence of misconduct in their particular field (e.g., Swazey, Anderson & Louis, 1993; Ranstam et al., 2000; Geggie, 2001; Martinson, Anderson & de Vries, 2005). Although direct questioning is a robust approach when gathering information on legitimate behaviours, when the topic is sensitive, estimates are subject to biases that reduce the validity of data (Macfarlane, Zhang & Pun, 2012); participants may either fail to respond (Groves, 2006) or bias answers towards socially acceptable positions (Fischer, 1993). Methods have therefore been developed specifically for estimating the prevalence of sensitive behaviours. These methods are intended to encourage more truthful reporting by providing respondents with levels of protection greater than simply guarantees of anonymity; critically, these methods make it impossible to directly link answers to individuals. However, just one of these methods has been used to investigate misconduct in academic research (List et al., 2001). Here we apply direct questioning and two specialised methods in order to estimate the prevalence of research misconduct amongst UK academics currently conducting research within biological sciences.

## Materials and Methods

The study focussed on UK-based academics engaged in research in the biological sciences. Our study was confined to academics based in departments offering undergraduate biology. The online questionnaire developed using SurveyGizmo (www.surveygizmo.com) was piloted before going live. Data collection covered a one-month period starting on 24th June 2013. Personalised emails introducing the authors, explaining the study and providing a URL link to the survey were sent to departmental heads and senior administrators of 59 university departments. One reminder was sent halfway through the study period. Ethical approval for the study was first received from the University of Kent, Unit for the Enhancement of Learning & Teaching’s ethics board (CSHE/MA/DR01).

The survey contained four sections: misconduct measured using the unmatched-count technique (UCT); misconduct measured using the crosswise-model (CM); academic background ranking of severity of misconduct; and misconduct measured using a direct question (DQ). Each method asked five sensitive questions associated with misconduct in research, two major (plagiarism, fabrication of data) and three minor (over-selling of results, inappropriate co-authorship, taking someone else’s idea).

### Unmatched-count technique

The UCT and its variants have been used to investigate many sensitive topics, including race (Kuklinski et al., 1997), health-risk behaviours (Hubbard, Caspar & Lessler, 1989) and illegal hunting (Nuno et al., 2013). UCT involves randomly assigning participants to one of two groups; the control and the treatment group. The control group are given a list of non-sensitive statements (e.g., statements associated with UK-based academia and research) and asked to report how many statements apply to them, without specifying which. The treatment group receives the same statements with the addition of one sensitive statement. For each misconduct question, respondents were randomly assigned to the control group or treatment group using a randomising function within SurveyGizmo. The proportion of the sample engaged in each sensitive behaviour was calculated as the difference in the mean number of statements between the control and treatment groups (Glynn, 2013).

### Crosswise-model

The CM, also developed for investigating sensitive topics (Yu, Tian & Tang, 2008), has not been widely applied. CM simultaneously asks respondents two questions, one non-sensitive and the other sensitive. Respondents state whether their answer is (a) Yes to both questions, or No to both questions, or (b) Yes to one question and No to the other. The non-sensitive question has to have a known probability (e.g., month of birth). In this study the non-sensitive question “*Is your birthday in month a, b or c*” was paired with one of five misconduct questions (Article S1). Birth months included in the non-sensitive question were selected at random. The proportion of the sample (*π*) involved in the sensitive behaviour is calculated as:

\begin{eqnarray*} \displaystyle \pi =\frac{\lambda +p-1}{2 p-1},\quad p\not = 0.5&&\displaystyle \end{eqnarray*}

where *λ* is the proportion of respondents that chose option (a) (Yes to both or no to both questions), and *p* is the proportion of the population that would answer yes to the non-sensitive question (Yu, Tian & Tang, 2008). Births per month are known to vary, so true monthly birth distributions were calculated from national data (ONS, 2013). Sample variance was estimated as:

\begin{eqnarray*} \displaystyle v a r(\pi )=\frac{\pi (1-\pi )}{n}+\frac{p(1-p)}{n(2 p-1)^{2}}&&\displaystyle \end{eqnarray*}

where *n* is the number of respondents (Jann, Jerke & Krumpal, 2012).

### Direct questioning

To explore the relative utility of UCT and CM compared with DQ, respondents were directly asked to indicate their involvement in each of the academic misconduct behaviours. The five behaviours were presented as a list and respondents were instructed to tick each activity that they had engaged in.

### Academic background

Respondents were asked a series of non-sensitive questions related to their academic background, research and ethics (Article S1). Respondents were also asked to rank the five misconduct behaviours in decreasing order of severity.

## Results

One hundred and eighty seven academics participated in the survey. Of these, 49.5% completed the entire questionnaire including the DQ, 54.5% completed the UCT and 52.4% completed the CM (no significant difference in results were detected between wholly completed and partially completed surveys). Over one third (36.6%) of respondents received most of their research funding from Research Councils UK; the same proportion reported receiving training in research ethics. However, 52.7% reported that their institution did not provide ethics training. Over 40% of respondents reported that grant proposals had to be seen by an ethics committee prior to submission. Respondents considered fabrication of data to be the most serious of the five misconduct behaviours (Table 1).

**Table 1:** Respondents’ ranking of unethical behaviours, 1 being the most serious and 5 being the least serious.

Behaviour	Average ranking	Agreement with ranking (%)
Fabricating data	1.6	76.3
Plagiarism	2.5	53.8
Taking someone else’s idea	3.1	43.7
Over-selling of results	3.6	38.3
Inappropriate co-authorship	4.3	62.9

For three of the five misconduct behaviours (plagiarism, over-selling of results, and inappropriate co-authorship) UCT gave the highest estimates of prevalence (Table 2). However, due to high variance, results for plagiarism and over-selling of results were statistically indistinguishable from estimates gained from DQ. DQ gave the highest estimates for taking other peoples’ ideas and fabrication of data (0%), however for the latter the UCT result was also statistically indistinguishable from zero. CM performed poorly with three of the five results giving negative estimates statistically less than zero.

**Table 2:** Prevalence of misconduct amongst biological sciences academics in the UK. Results are presented as a percentage of the sampled population (±SE) for the unmatched-count technique (UCT), crosswise-model (CM) and direct questioning (DQ). The method producing the highest estimate is highlighted in bold and results are presented in declining order of the severity of the misconducts (Table 1).

Method	UCT	CM	DQ
Fabricated	−4.7 (±12.0)	−5.0 (±0.9)	**0.0**
Plagiarised	**4.2 (±10.8)**	−2.4 (±0.8)	1.1
Taken idea	−32.2 (±13.5)	−15.0 (±0.7)	**1.1**
Over-sold	**25.3 (±13.8)**	13.5 (±0.9)	8.6
Co-authored	**68.7** (±12.2)	23.3 (±1.0)	29.0

Prevalence of misconducts largely followed respondents’ rankings of the seriousness of the behaviours. Inappropriate co-authorship was ranked the least serious issue and was the most prevalent (68.7%, UCT), whereas fabrication of data, ranked the most serious, was the least prevalent at 0.0% (DQ) (see Article S2 for discussion of methodologies and Table S3 for the raw data output).

## Discussion

Fabrication, falsification and plagiarism (FFP) are acknowledged as being the most serious forms of research misconduct. Consequently, some studies have considered these categories together. In a meta-analysis of scientific misconduct, Fanelli (2009) found that estimates of fabrication and falsification ranged from 0.3 to 4.9% with a weighted mean of 1.97%. Our analysis yielded values of 0.0% and 1.1% for fabrication of data and plagiarism respectively when based on direct questioning. However, when UCT was used, the estimated prevalence of plagiarism rose to 4.2%. This result is comparable with that of another study that used a specialised questioning technique; List et al. (2001) used the randomised response technique (RRT) and found 4.5% falsification by economists.

Most studies of research misconduct have focussed on cases of FFP; few have investigated less serious forms of misconduct. In this study we estimated the prevalence of taking other people’s ideas, over-selling results, and inappropriate co-authorship. Our DQ estimate of taking of other people’s ideas (1.1%) is comparable with those in other studies (e.g., Martinson, Anderson & de Vries, 2005). However our UCT and CM estimates for this behaviour were both negative. Reasons for negative UCT estimates include the small sample size, the number of statements, and the relationships between statements included on the lists (for further discussion see Article S2). Negative CM estimates may be due to respondents feeling insufficiently protected, as the pairs of questions consist of one non-sensitive question (month of birth) that is unrelated to the sensitive question. We estimated that at least 68% (UCT) of researchers inappropriately co-authored papers. Using DQ, others have estimated that inappropriate co-authorship occurs amongst just 31% to 37% of researchers (Swazey, Anderson & Louis, 1993; Geggie, 2001).

Results of our comparative study provide evidence that DQ can be subject to considerable under-reporting when the topic of investigation is sensitive. For example, only 29% of respondents admitted to inappropriate co-authorship when asked via a DQ whereas over 68% admitted to this behaviour via UCT. List et al. (2001) used DQ and a specialised questioning technique (RRT) to look at four minor infractions amongst economists, including inappropriate co-authorship. RRT gave an estimated prevalence of 10%, compared with 7.5% for DQ. Assuming that the RRT estimate approximates the prevalence of inappropriate co-authorship amongst economists (it is likely to be lower since List et al. (2001) investigated four infractions together), their estimate is considerably lower than those found in this study using either UCT or DQ. This could potentially be due to greater collaboration in the biological sciences compared with economics, but may also reflect other factors such as ‘gatekeepers’ and the access to biological information and facilities. No other study has examined the prevalence of over-selling of results; a behaviour that we estimate could be prevalent in up to one-quarter of academics in the biological sciences.

In summary, questions relating to research misconduct are sensitive even when the form of the misconduct appears widespread. While norms, such as including those who act as gatekeepers on papers, may explain the high prevalence of inappropriate co-authorship, the fact remains that the UCT estimate was over twice that of DQ. This clearly illustrates that even forms of misconduct that are perceived to be minor, and arguably are becoming the norm, are still subject to under-reporting. Estimates from previous studies, most of which used some form of DQ, should therefore be considered under-estimates of the true prevalence rates. This and other studies suggest that known cases are just the “tip-of-the-iceberg”. Many incidences of research misconduct pass unreported and, in some cases, the type of misconduct goes unrecognised.

## Supplemental Information

10.7717/peerj.562/supp-1 Article S1 Study questionnaire with brief explanatory notes

10.7717/peerj.562/supp-2 Article S2 Comparing different methodologies for asking sensitive questions

10.7717/peerj.562/supp-3 Table S3 Table S3 Raw data output from questionnaire
