# Supplementary material for: Estimating the prevalence of researcher misconduct: a study of UK academics within biological sciences
Source: PeerJ. 2014 Sep 9;2:e562. doi: 10.7717/peerj.562 (PMC4168756; doi:10.7717/peerj.562)
Supplement: Article S1 [file peerj-02-562-s001.pdf]

Supplementary Information

Article S1: Study questionnaire with brief explanatory notes.

Initial statement

The following introduction was given at the beginning of the questionnaire and a similar introduction was provided to heads of departments and senior administration managers. By continuing the survey consent was implied.

"Welcome to my survey looking at ethical behaviour in academic research. This survey is part of a research project towards my MA in Higher Education, therefore your completing this survey is very much appreciated. As the title suggests, the questionnaire is looking at the ethical behaviour of those undertaking research in academia, specifically those that hold academic positions. The questionnaire is completely anonymous and should take no more than 3-4 minutes of your time."

Unmatched-count technique list of statements including one sensitive statement (highlighted in italics) for each of the five sensitive statements under investigation; the control lists being the same as the treatment minus the sensitive statement. Participants were asked to provide a value between 0 and 7 for the control or 0 and 8 for the treatment.

1) How many of the following statements do you agree with/have done?

- I conduct research myself
- Last year I published fewer than 3 papers

- Last year I received funding from one of the UK Research Councils
- *I have plagiarised other peoples' work*
- My university provides training specifically on Ethics in Research
- Most of my research is lab-based
- I have attended a training session in research ethics
- Before submitting a grant proposal it has to be passed by an ethics committee

2) How many of the following statements do you agree with/have done?

- I have a senior admin role within the school/dept
- My admin role does not impact on my research
- I always get a colleague to independently read over my grant proposal
- I currently supervise fewer than 5 PhD students
- All our PhD students must have at least 2 supervisors
- UK Border Agency has not had a negative impact on postgraduate research
- *I have over-sold the results in a paper*
- As part of postgraduate research training all students are required to complete a course on research ethics

3) How many of the following statements do you agree with/have done?

- I convene a postgraduate module
- I generally do not accept students who come to me with their own research projects
- *I have been a co-author on a paper when I have done little to warrant my inclusion*
- I feel like I have little time to undertake research
- The move by research councils to large consortium grants is a positive step
- I hold formal meetings with my research group at least every two weeks

- I do not find it difficult to juggle work/private life balance
- I subscribe to Times Higher Education

4) How many of the following statements do you agree with/have done?

- Most of my research students have come to work with me through contacts rather than through a response to adverts
- I am happy with the level of funding the government puts into scientific research through the Research Councils
- I do not believe it is becoming more difficult to get research papers accepted by peer-reviewed journals
- I believe the loss of NERC's small grant rounds will have a negative impact on my own research
- My research field sites are mainly outside the UK and EU
- I have at least one post doc in my research group
- *I have fabricated data and then published the results*
- I am worried that the Freedom of Information Act will impact my research

5) How many of the following statements do you agree with/have done?

- I am a member of the NERC review college
- I do not believe it is becoming more difficult to get research grants from UK Research Councils
- I believe all research papers should be open-access
- I believe all data should be made available online within 6 months of a paper being published
- I have been on a media training course

- *I have taken a research idea I heard at a meeting or conference and published it as my own?*
- Young investigators require more funding opportunities from the UK Research Councils
- I always try to include junior investigators on grant applications as co-PIs to help their career

Crosswise-model questions containing one non-sensitive question with a known frequency (indicated in brackets after the question but not provided to the participants) and one sensitive question.

1) Please read the following two questions

Q1: Is your birthday in January, March or April? (0.247)
Q2: Have you ever plagiarised other peoples' work?

Now select your response
( ) My response is NO to both questions OR YES to both questions
( ) My response is YES to one of the question AND NO to the other

2) Answer the following two questions

Q1: Is your birthday in August, November or December? (0.249)
Q2: Have you ever over-sold the results in a paper?

Now select your response

( ) My response is NO to both questions OR YES to both questions

( ) My response is YES to one of the question AND NO to the other

3) Answer the following two questions

Q1: Is your birthday in January, April or September? (0.250)

Q2: Have you ever fabricated data and then published the results?

Now select your response

( ) My response is NO to both questions OR YES to both questions

( ) My response is YES to one of the question AND NO to the other

4) Answer the following two questions

Q1: Is your birthday in June, August or December? (0.252)

Q2: Have you ever been a co-author on a paper when you have done little to warrant inclusion?

Now select your response

( ) My response is NO to both questions OR YES to both questions

( ) My response is YES to one of the question AND NO to the other

5) Answer the following two questions

Q1: Is your birthday in February, June or November? (0.241)
Q2: Have you ever taken a research idea you heard at a meeting or conference and published it as your own?

Now select your response
( ) My response is NO to both questions OR YES to both questions
( ) My response is YES to one of the question AND NO to the other

Questions asked in the study to gain and understanding of the participant's position within academia, research and their engagement with research ethics.

1. In what year were you born?
2. In which country did you do your first degree (e.g. BSc)? (selected from a list of countries)
3. What is your current position? (selected from Post doc/Research Fellow, Lecturer, Senior Lecturer, Reader, or Professor)
4. In which year did you publish your first peer-reviewed paper?
5. How many papers do you publish each year? (selected from 2 or less, 3-5, 6-9, 10-14, 15 or more)
6. Does most of your funding come from UK Research Councils? (selected from yes or no)
7. Does your university or institute provides training specifically on Ethics in Research? (selected from yes, no or don't know)

8. Have you attended a training session on research ethics? (selected from yes or no)
9. Before submitting a grant proposal are you required to have it be passed by an ethics committee? (selected from yes or no)

Direct questions.

Have you ever... (select all that apply)

- plagiarised other peoples' work?
- over-sold the results of a paper?
- fabricated data and then published the results?
- been a co-author on a paper when you have done little to warrant inclusion?
- taken a research idea you heard at a meeting or conference and published it as your own?
