# Supplementary material for: Estimating the prevalence of researcher misconduct: a study of UK academics within biological sciences
Source: PeerJ. 2014 Sep 9;2:e562. doi: 10.7717/peerj.562 (PMC4168756; doi:10.7717/peerj.562)
Supplement: Article S2 [file peerj-02-562-s002.pdf]

Article S2: Comparing different methodologies for asking sensitive questions

The results of this study show that the UCT outperformed the CM in each of the five behaviours studied, giving higher levels of prevalence. However, UCT suffered from high variance, potentially due to the large number of statements included on each UCT list and lack of negative correlation between statements. Reducing list length and increasing negative correlation between statements may have reduced both the variance and presence of ceiling effects (Glynn, 2013). The CM suffered from negative values that were significantly different from zero or gave significantly lower values than the estimate gained by direct questioning; only in the case of over-selling did the CM give significantly higher estimates than DQ. This may be due to one or more of several reasons, such as the sample population differing from the population for the distribution of the non-sensitive question (i.e. month of birth).

Unmatched-count technique

The selection of statements used in the UCT is important. In an attempt to reduce floor and ceiling effects (responding to all statements positively or negatively to all statements respectively, thereby removing any protection for the participant), it is recommended that a mixture of both high- and low-prevalence statements is used (Droitcour et al., 1991; Tsuchiya, Hirai & Ono, 2007). Further, to increase protection, the list of statements needs to be sufficiently long to again reduce floor and ceiling effects (Kuklinski, Cobb & Gilens, 1997); however lengthening the list typically results in increased variance (Tsuchiya, Hirai & Ono, 2007; Corstange, 2009) and also impacts on whether the participant can remember their responses, thereby introducing measurement bias (Tsuchiya, Hirai & Ono, 2007). As a result, researchers are left with a dilemma, on the one hand wishing to reduce bias from floor and ceiling effects and on the other variance. It is possible to reduce the impact of these trade-offs by choosing statements that are negatively correlated; that is, if a participant agrees with one

statement they are highly unlikely to agree with the other (Glynn, 2013). Within this study, although attempts were made to reduce floor and ceiling effects by having both low and high prevalence statements, it is clear that this was not entirely successful, particularly for the floor effect (in two of the questions the floor effect was between 11% and 13%). Furthermore, although some attempt was made to generate negatively correlated statements, this again had limited success, standard errors reaching between 10% and 14%. This is likely due to the length of the lists for each question, as each question had seven statements and therefore in total 35 higher education and/or research related statements had to be generated. This resulted in the potential for greater variance, which may have been reduced if four or five statements for each question had been given as the range of possible answers to the question. Also, given the number of statements required, negatively correlated statements were unlikely to be optimal. Any future study should consider using only four or five non-sensitive statements per list. In the study presented here, of 187 participants who began the survey, only 52.4% completed the UCT questions. This relatively low response rate also impacts on estimating the prevalence of a behaviour by producing larger standard errors. In a study of bushmeat hunting, 1191 individuals interviewed using the UCT, of which only 28 refused to take part; Nuno et al. (2013) estimated prevalence of bushmeat hunting as 18%, with a standard error of 5%. Their study used only four non-sensitive questions.

Crosswise-model

Although this method has been tested empirically, it is sufficiently new that further research is required to evaluate its utility. The method is efficient, with low variance, but the estimates of prevalence were unrealistic in that they generated values that were negative or less than those generated by the direct question in all but one case. Interestingly, the two behaviours that resulted in positive estimates were also the two that were ranked as the least serious by

participants. As mentioned in the Materials and Methods section, the proportion of the sample (π) involved in the sensitive behaviour is calculated as:

$$\pi = \frac{\lambda + p - 1}{2p - 1}, p \neq 0.5$$

where λ is the proportion of the respondents that chose option (a) (i.e. Yes to both or No to both questions), and p is the proportion of the population that would answer Yes to the non-sensitive question (Yu, Tian & Tang, 2008). Therefore, if no-one in the population was engaged in the behaviour then all individuals in the population would be saying No to the sensitive question, and p would be saying No to the non-sensitive question. Therefore, λ would equal 1-p and the proportion of the sample (π) involved in the sensitive behaviour would equal zero. If π is less than 0, then λ has to be greater than 1-p. For this to occur in the case of the three months of birth, the proportion of the participants who would answer Yes to the non-sensitive question has to be less than that of the true population on which p is based. That is, in the case of this study, the months of birth of research academics is not randomly distributed, fewer being born in the months of the non-sensitive question than expected. For example, the sensitive question on fabrication generated a negative value of -4.8% (±0.9) to -5.0% (±0.9) compared with 0.0% from direct questioning (Table 2). The non-sensitive question was "*Is your birthday in January, April or September?*" Based on national statistics, as discussed in the methods section, the proportion of the population that have a birthday in one of these three months is 0.24965. Assuming none of the participants were engaged in data fabrication as indicated by the direct questioning, then to achieve a score of -4.8% to -5.0%, the proportion of the respondents with a birthday in one of the three months must be 0.20465 to 0.20277. This is 18.0% to 18.8% lower than the estimated proportion for the population. With a difference between the participant population and the national population in terms of

months of birth, based on the incorrect assumption that $p = 0.24965$ (rather than 0.20465 to 0.20277), data fabrication could be as high as 4.68% and CM would still would give a prevalence estimate of zero.

Various attributes, including career and intelligence, have been suggested to be linked to the month of birth (Crawford, Dearden & Meghir, 2007; Department for Education, 2010; Crawford, Dearden & Greaves, 2011), which could explain the results seen here although the months were chosen at random to try to reduce this possibility. However, another possibility is that for some reason participants preferred to answer "*NO to both questions OR YES to both questions*" rather than "*YES to one of the questions AND NO to the other*." Participants may have felt less protected if they answered "*YES to one of the questions AND NO to the other*" in that it is perceived to have opened the possibility that they have been involved in data fabrication. This is a reasonable assumption since the results of the CM increase in reliability in terms of being equal to or greater than the result of direct questioning with decreasing seriousness of the behaviour (Table 2). With UCT, where the sensitive statement is hidden among a list of non-sensitive questions, the participant may not know the purpose of the study other than the general topic, therefore they may be more open to admitting to a sensitive statement in this more protected environment than with the CM; if so this would raise potential ethical issues regarding consent. With the CM, one has a non-sensitive question, which in this case was not even vaguely related to the sensitive question. Since the non-sensitive question requires a known proportion in this case, the proportion of people born in one of three months; it is difficult to envisage a non-sensitive research integrity or ethics question for which the proportion of the population is known. Therefore the sensitive question of the CM is more obvious and may trigger a sensitive response, resulting in a biased answer.

Besides the two reasons discussed, non-random distribution of the participants in respect to the non-sensitive question, and/ or the biased selection of the statement "*NO to both questions OR YES to both questions*", a third possibility exists, which is that participants failed to follow the instructions with sufficient care. This could potentially impact on the results, given that nearly 8% of participants ranked data fabrication as the least heinous crime, compared with 76.3% ranking it as the most serious (14.1% ranked it in the bottom three of five). This discrepancy may be considered unusual, given that it is one of the three forms of misconduct (i.e. FFP) highlighted by most organisations involved in academic integrity.

REFERENCE

Corstange D. 2009. Sensitive questions, truthful answers? Modeling the list experiment with LISTIT. *Political Analysis* 17:45-63.

Crawford C, Dearden L, Meghir C. 2007. *When you are born matters: the impact of date of birth on child cognitive outcomes in England*. Centre for the Economics of Education Report, Department for Children, Schools and Families, UK.

Crawford C, Dearden L, Greaves E. 2011. *The impact of month of birth on children's cognitive and non-cognitive skills in England*. Institute for Fiscal Studies, UK.

Department for Education. 2010. *Month of birth and education*. DfE Research Report 17, UK.

Droitcour J, Caspar RA, Hubbard ML, Parsley TL, Visscher W, Ezzati TM. 1991. The item count technique as a method of indirect questioning: A review of its development and a case study application. In: Biemer PP, Groves RM, Lyberg LE, Mathiowetz N, Sudman S,

eds. *Measurement errors in surveys*. New York: John Wiley & Sons, 185-210.

Glynn A. 2013. What can we learn with statistical truth serum? Design and analysis of the list experiment. *Public Opinion Quarterly* 77:159-172.

Kuklinski JH, Cobb MD, Gilens M. 1997. Racial attitudes and the “New South”. *The Journal of Politics* 59:323-349.

Nuno A, Bunnefeld N, Naiman L, Milner-Gulland. 2013. A novel approach to assessing the prevalence and drivers of illegal bushmeat hunting in the Serengeti. *Conservation Biology* 27:1355-1365.

Tsuchiya T, Hirai Y, Ono S. 2007. A study of the properties of the item count technique. *Public Opinion Quarterly* 71:253-272.

Yu J-W, Tian G-L. Tang M-L. 2008. Two new models for survey sampling with sensitive characteristic: design and analysis. *Metrika* 67:251-263.
